# The Contribution of Transference Focused Psychotherapy in Improving Psychiatry Trainees’ Attitude and Technical Confidence Towards Patients With Personality Disorders

**DOI:** 10.1192/bjo.2022.97

**Published:** 2022-06-20

**Authors:** Arianna Sinisi, Mattia Marchi, David Prior, Tennyson Lee

**Affiliations:** 1 Department of Biomedical, Metabolic and Neural Sciences, University of Modena and Reggio Emilia, Modena, Italy; 2 Centre for Understanding Personality Disorder, London, United Kingdom; 3 Deancross Personality Disorder Service, East London Foundation Trust, London, United Kingdom

## Abstract

**Aims:**

Transference-focused psychotherapy (TFP) is a manualized evidence-based treatment for severe personality disorders (PDs) based on a psychodynamic approach that focuses on object relations theory. It has been used as a teaching tool in different psychiatric settings. Psychiatry trainees are often the “first-responders” in multiple services, and they have to deal with patients with PDs in various settings. Yet there is a documented gap in psychiatry trainees’ education regarding the assessment and management of patients with PD pathology. The aim of our study was to evaluate whether a series of teaching sessions on TFP theory and techniques as applied to PD could improve the attitude and technical confidence of psychiatric trainees in the clinical encounter of a patient with a PD.

**Methods:**

Two cohorts of psychiatry trainees in Tower Hamlet's East London Foundation Trust received four teaching sessions, each of one hour duration, on TFP theory and techniques. All the sessions were delivered online, using video teleconferencing software. 14 Trainees completed 2 questionnaires, pre- and post-teaching: the Attitude to Personality Disorder Questionnaire (APDQ) and the Clinical Confidence with Personality Disorder Questionnaire (CCPDQ). The APDQ asks the responder to score from 1–6 the frequency they experience certain feelings towards patients with PD. In the absence of a suitable instrument, we developed the CCPDQ consisting of a set of 13 questions rated on a 6-point Likert scale addressing key issues identified in TFP including establishing and maintaining the treatment frame and in implementing the 4 main techniques. We also conducted a 1-hour focus group post teaching which was videorecorded, transcribed, and analysed thematically.

**Results:**

On quantitative analysis, the Wilcoxon signed-rank test indicated statistically significant improvements in the total APDQ score (P = 0.003, r = 0.81) and in the CCPDQ questionnaires (P = 0.001, r = 0.88).

The thematic analysis showed an overall positive effect of the TFP teaching on trainees’ attitude and confidence: they felt it improved their understanding of the nature of personality disorder, their awareness and management of countertransference, awareness of object relations and relation dyads at play in the encounter.

**Conclusion:**

Training junior doctors about TFP theory and techniques as applied to PD can significantly improve their attitude towards these patients and their technical confidence in the clinical encounter. Of note, our workshop is resource light and can easily be delivered by remote teaching. Based on these findings, teaching of TFP in the core psychiatric training curriculum should be considered.

